# The eIF4E-Binding Protein Eap1p Functions in Vts1p-Mediated Transcript Decay

**DOI:** 10.1371/journal.pone.0047121

**Published:** 2012-10-10

**Authors:** Laura M. Rendl, Melissa A. Bieman, Heli K. Vari, Craig A. Smibert

**Affiliations:** 1 Department of Biochemistry, University of Toronto, Toronto, Ontario, Canada; 2 Department of Molecular Genetics, University of Toronto, Toronto, Ontario, Canada; University of Cambridge, United Kingdom

## Abstract

Sequence-specific RNA binding proteins can induce the degradation of mRNAs through their ability to recruit proteins that trigger transcript destabilization. For example, Vts1p, the *S. cerevisiae* member of the Smaug family of RNA binding proteins, is thought to induce transcript decay by recruiting the Ccr4p-Pop2p-Not deadenylase complex to target mRNAs. The resulting deadenylation triggers transcript decapping followed by 5′-to-3′ exonucleolytic decay. Here we show that the eIF4E-binding protein, Eap1p, is required for efficient degradation of Vts1p target transcripts and that this role involves the ability of Eap1p to interact with eIF4E. Eap1p does not stimulate deadenylation of Vts1p target transcripts but is instead involved in decapping. Eap1p interacts with Vts1p and mediates an indirect interaction between Vts1p and eIF4E. Taken together these data suggest a model whereby the interaction of Vts1p with Eap1p at target mRNAs stimulates decapping.

## Introduction

Regulation of mRNA degradation has an important role in the control of gene expression. In *Saccharomyces cerevisiae* the major mRNA decay pathway is initiated through transcript deadenylation mediated by the Ccr4p-Pop2p-Not complex [1], [2], [3]. After deadenylation the transcript is decapped by a heterodimeric complex composed of Dcp1p and Dcp2p (reviewed in [4], [5]). In yeast numerous factors that positively regulate mRNA decapping have been identified including Pat1p, Dhh1p, Edc1p, Edc2p, Edc3p and the Lsm 1-7 complex (reviewed in [4], [5]). After decapping the body of the transcript is degraded 5′-to-3′ by the exonuclease Xrn1p [2], [6].

Sequence-specific RNA binding proteins can add another level of control to the regulation of mRNA stability [7]. Typically these proteins bind mRNA target sequences and interact with other trans factors that influence the rate of mRNA decay. The Smaug (Smg) family of post-transcriptional regulators, which are conserved from yeast to humans, bind RNA through a conserved sterile alpha motif (SAM) domain that interacts with stem-loop structures termed Smg recognition elements (SREs) [8], [9], [10], [11], [12], [13], [14], [15], [16], [17]. Vts1p, the Smg family member in *S. cerevisiae*, stimulates mRNA degradation through deadenylation by the Ccr4p-Pop2p-Not deadenylase complex [12], [18]. Following deadenylation Vts1p target transcripts are decapped and then degraded by the 5′-to-3′ exonuclease Xrn1p [18]. A similar mechanism of deadenylation-dependent mRNA decay is employed by Smg in *Drosophila* [15], [17], [19]. Both Vts1p and Smg interact with the Ccr4p-Pop2p-Not complex suggesting a model whereby these proteins induce transcript decay by recruiting the deadenylase to target mRNAs. Smg also regulates mRNA translation through a separate mechanism involving an interaction with the eIF4E-binding protein Cup [20]. Cup binds to the mRNA cap binding protein eIF4E through a canonical eIF4E-binding motif (YXXXXLΦ, where Φ is a hydrophobic amino acid). Cap-dependent translation initiation involves eIF4E recruiting eIF4G to an mRNA, which indirectly mediates recruitment of the 40S ribosome [21]. eIF4G also interacts with eIF4E through an eIF4E-binding motif and thus recruitment of Cup to an mRNA inhibits translation by blocking the eIF4E/eIF4G interaction [20], [22].

The role of Cup in Smg function led us to speculate that Vts1p might also regulate target mRNAs through an eIF4E-binding protein. While there is no Cup homolog in yeast, two eIF4E-binding proteins, Caf20p and Eap1p, have been identified [23], [24], [25]. In addition, global genetic analysis revealed synthetically lethal interactions between Eap1p and two deadenylase components, Ccr4p and Pop2p [26], suggesting a functional relationship, either direct or indirect, among the gene products. This genetic interaction combined with the role of the Ccr4p-Pop2p-Not deadenylase in Vts1p-mediated regulation prompted us to test if Eap1p might function with Vts1p to regulate target mRNAs. Using two different Vts1p target mRNAs we demonstrate that Eap1p is required for efficient Vts1p-mediated transcript degradation. Eap1p does not stimulate deadenylation but is instead required for efficient removal of the 5′ cap. In addition, Eap1p-mediated stimulation of transcript decay requires binding to eIF4E. We also find that Eap1p biochemically interacts with Vts1p and is able to mediate an indirect interaction between Vts1p and eIF4E. Taken together these data suggest a model whereby the Vts1p/Eap1p/eIF4E complex stimulates transcript decapping.

## Results

### Eap1p is Required for Efficient Decay of Vts1p Target mRNAs

**Figure 1:**
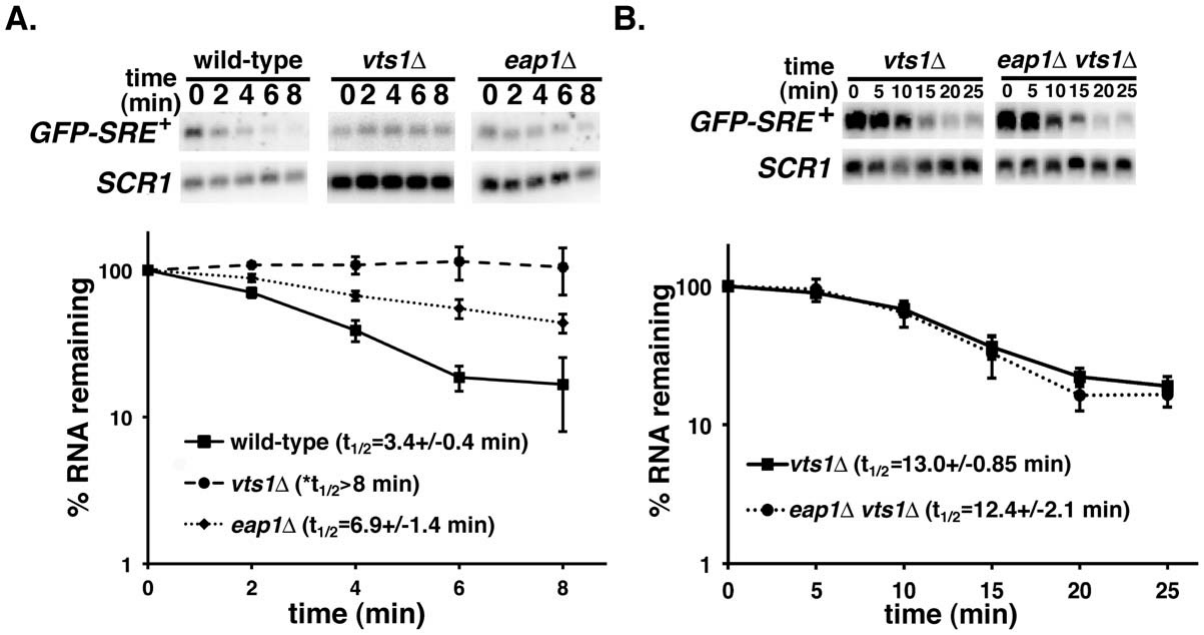
Eap1p and Vts1p function in the same pathway to destabilize *GFP-SRE^+^* mRNA. *GFP-SRE^+^* mRNA expression was induced in the indicated strains and then shut-off with glucose and reporter mRNA levels were assayed at the times indicated after transcriptional shutoff by Northern blot. The results of at least three independent experiments were quantitated and normalized using the levels of *SCR1* RNA and graphed with error bars representing standard deviation. *Note that an accurate measure of the half-life of *GFP-SRE^+^* mRNA can be found in Figure S2.

To assess the role of Eap1p in Vts1p function we first examined the stability of a reporter mRNA which recapitulates Vts1p-mediated decay *in vivo* [12]. The GFP-SRE*^+^* reporter encodes green fluorescent protein (GFP) under the control of the inducible galactose promoter and has three SREs in its 3′ untranslated region (UTR). A transcriptional pulse-chase approach was used to measure the stability of reporter mRNAs by Northern blot after transcriptional induction by galactose and subsequent repression by the addition of glucose. We previously reported that *GFP-SRE^+^* mRNA is rapidly degraded in wild-type cells while it is stabilized in a *vts1Δ* strain [18]. Here we show that rapid degradation of *GFP-SRE^+^* mRNA was compromised in *eap1Δ* cells (Figure 1A). The fact that the *GFP-SRE^+^* mRNA was stabilized more in a *vts1Δ* strain than in an *eap1Δ* strain suggests that while Eap1p plays a role in the decay of this mRNA it is not absolutely required for Vts1p function.

These data could suggest that Eap1p functions in the same pathway or a separate pathway to regulate the stability of *GFP-SRE^+^* mRNA. To differentiate between these two possibilities we compared *GFP-SRE^+^* mRNA in *vts1Δ* cells and *eap1Δ vts1Δ* double delete cells (Figure 1B) and found that this mRNA has the same stability under these different conditions. This suggests that Vts1p and Eap1p function together in the same pathway to degrade *GFP-SRE^+^* mRNA.

**Figure 2:**
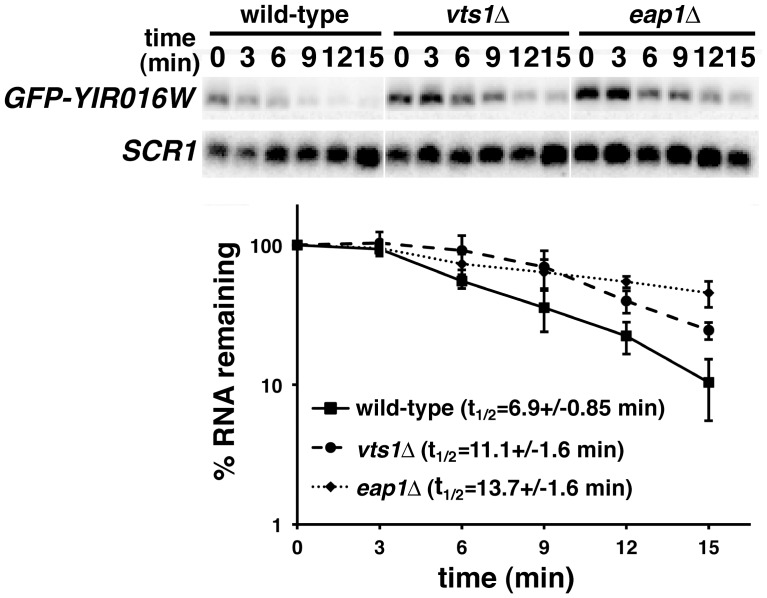
Eap1p is required for the destabilization of *GFP-YIR016W* mRNA. mRNA expression was induced in the indicated strains and then shut-off with glucose and reporter mRNA levels were assayed at the times indicated after transcriptional shutoff by Northern blot. The results of at least three independent experiments were quantitated and normalized using the levels of *SCR1* RNA and graphed with error bars representing standard deviation. Note that the half-life of *GFP-YIR016W* mRNA is significantly increased in both *vts1Δ* and *eap1Δ* cells as judged by a t test (P<0.01).

To further confirm the importance of Eap1p in the degradation of Vts1p target mRNAs we measured the stability of *YIR016W* mRNA in *eap1Δ* cells, having previously shown that Vts1p binds to this mRNA and regulates its stability through deadenylation, decapping and 5′-to-3′ exonucleolytic decay [12], [18]. To do this we used a reporter construct in which GFP is fused to the *YIR016W* ORF under the control of the GAL1 promoter (GFP-YIR016W). This construct allows us to perform transcriptional pulse/chase experiments similar to those described for the *GFP-SRE^+^* reporter and the GFP tag allows us to specifically detect this transcript in cells that contain endogenous *YIR016W* mRNA. We induced GFP-YIR016W reporter transcription by adding galactose to *eap1Δ* cells and then shut off transcription with glucose. Similar to our findings using the *GFP-SRE^+^* reporter, we found that the stability of *GFP-YIR016W* mRNA was increased in the *eap1Δ* strain as compared to wild-type (Figure 2). Taken together these data indicate that Eap1p is required for the rapid decay of Vts1p target mRNAs.

**Figure 3:**
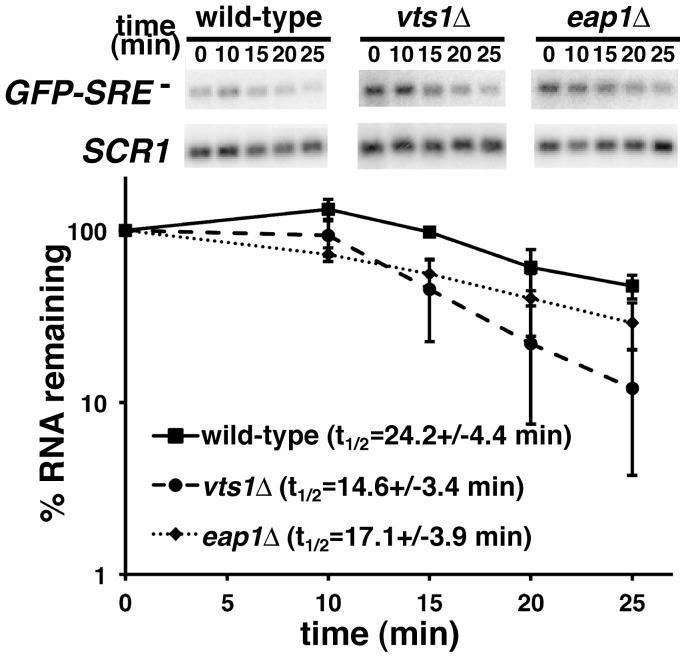
Eap1p is not required for the degradation of *GFP-SRE* *^-^* **mRNA.** GFP-SRE*^-^* gene transcription was induced in wild-type, *vts1Δ* and *eap1Δ* cells with galactose and then shut off with glucose and reporter mRNA levels were assayed at the times indicated after transcriptional shutoff by Northern blot. The results of at least two independent experiments were quantitated and normalized using the levels of *SCR1* RNA and graphed with error bars representing standard deviation.

The role of Eap1p in the degradation of Vts1p target mRNAs could indicate a general role in the degradation of mRNAs. Alternatively, its role could be more specific, perhaps reflecting a direct function in Vts1p-mediated decay. To explore these possibilities we assessed the stability of a GFP reporter mRNA (*GFP-SRE^-^*) which is identical to the *GFP-SRE^+^* reporter with the exception that it carries SREs in which the loop sequences are mutated to block Vts1p binding [12] and as such this mRNA is not destabilzed by Vts1p (Figure 3). Transcriptional pulse-chase experiments demonstrated that *GFP-SRE^-^* mRNA was not stabilized in *eap1Δ* cells and, in fact, the earlier time points suggest a modest destabilization of the mRNA in these cells (Figure 3). Similar to Vts1p target mRNAs [18], the *GFP-SRE^-^* mRNA was destabilized through the major mRNA decay pathway as degradation required Ccr4p (the catalytic subunit of the Ccr4p-Pop2p-Not deadenylase) and the 5′-to-3′ exonuclease Xrn1p (Figure S1). Thus, the differential role of Eap1p in the stability of *GFP-SRE^+^* and *GFP-SRE^-^* mRNAs is consistent with a direct role for Eap1p in the degradation of Vts1p target mRNAs as opposed to a general role in transcript degradation.

Interestingly, these experiments demonstrated that *GFP-SRE^-^* mRNA was less stable in a *vts1Δ* strain compared to wild-type cells (Figure 3). We suggest that the physical interaction between Vts1p and the Ccr4p-Pop2p-Not deadenylase complex [18] in wild-type cells sequesters some fraction of the deadenylase into a pool that is unable to act on mRNAs that are not targeted by Vts1p. In a *vts1Δ* strain this pool is no longer sequestered and as such is free to act on the *GFP-SRE^-^* mRNA, thereby accelerating its deadenylation and subsequent degradation. Consistent with this highly speculative model we see a similar stability for *GFP-SRE^+^* and *GFP-SRE^-^* mRNAs in *vts1Δ*cells in transcriptional pulse-chase experiments (Figure S2).

### Eap1p is not Required for Vts1p-mediated Deadenylation

We next set out to identify at which step in the mRNA decay process Eap1p functions. Given that Vts1p triggers mRNA decay via poly(A) tail shortening, mediated by the Ccr4p-Pop2p-Not deadenylase complex, we analyzed the poly(A) tail length of *GFP-SRE^+^* mRNA in *eap1Δ* cells to determine whether Eap1p was required for deadenylation of Vts1p target mRNAs. We used an RNase H cleavage method to measure the poly(A) tail length of the *GFP-SRE^+^* mRNA over an 8-min transcriptional pulse-chase time course. Total RNA samples from each time point were treated with RNase H in the presence of a specific antisense oligonucleotide that hybridized to the GFP open reading frame. After RNase H cleavage, the poly(A) tail of the resulting short 3′-end fragment of the GFP reporter mRNA was measured by high resolution polyacrylamide electrophoresis and Northern blotting. To one sample specific oligonucleotide and oligo(dT) were added and, as such, treatment with RNase H yielded a 3′-end fragment lacking a poly(A) tail, which served as a marker for the deadenylated 3′-end fragment.

**Figure 4:**
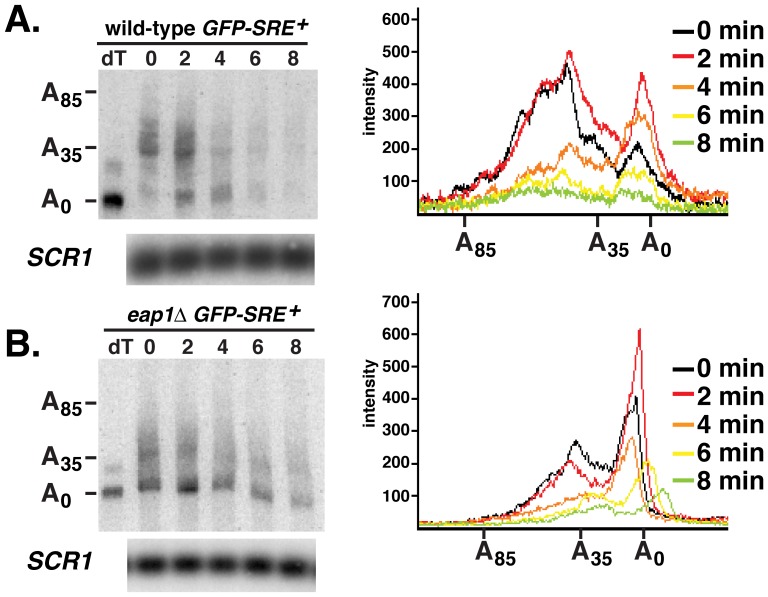
Eap1p is not required for transcript deadenylation. *GFP-SRE^+^* reporter transcription in wild-type cells (**A**) and *eap1Δ* cells (**B**) was induced with galactose and then shut off with glucose and an RNase H cleavage assay was used to measure the poly(A) tail length of the reporter transcript at the times indicated after transcriptional shutoff. Where indicated oligo(dT) was added to remove the poly(A) tail by RNase H treatment, providing a marker for deadenylated mRNA (dT). The distribution of poly(A) tail lengths at the indicated times after transcriptional shutoff are displayed on the graphs. An aliquot of each RNA sample was resolved in a separate Northern analysis and probed for *SCR1* RNA to serve as a control for loading and RNA integrity.

Comparison of the RNase H cleavage data from wild-type cells and *eap1Δ* cells (Figure 4) indicate that Eap1p is not required for deadenylation of the *GFP-SRE^+^* mRNA. For example, wild-type cells at time zero showed two populations of *GFP-SRE^+^* mRNA. One population had tail lengths from 30 to 50 nucleotides while the other had very short tails presumably representing transcripts that were deadenylated during the 16-min induction with galactose indicating the transcript was being rapidly deadenylated. At time zero *eap1Δ* cells also showed these same two populations of *GFP-SRE^+^* mRNA, again suggesting that this mRNA is rapidly deadenylated. In fact, *eap1Δ* cells show an increase in the fraction of deadenylated *GFP-SRE^+^* mRNA suggesting that the mRNA is being more rapidly deadenylated during the 16 minute galactose induction. In addition, in both wild-type and *eap1Δ* cells soon after the addition of glucose we saw a decrease in the amount of longer poly(A) tail species and an increase in the amount of the shorter tail population again indicating no deadenylation defect in *eap1Δ* cells. At later time points deadenylated species persisted in the *eap1Δ* cells, similar to results we reported for mutants that are wild-type for deadenylation but defective for downstream steps such as decapping and 5′-to-3′ exonucleolytic decay [18]. Taken together these data suggest that Eap1p is not required for Vts1p-mediated deadenylation and if anything it might antagonize this process. Thus, the stabilization of *GFP-SRE^+^* mRNA in *eap1Δ* cells does not result from a defect in deadenylation but instead must reflect a role for Eap1p in a subsequent step of the decay process.

### Eap1p Stimulates mRNA Decapping

After deadenylation Vts1p target transcripts are decapped and then degraded in a 5′-to-3′ direction by the exonuclease Xrn1p [18]. To determine if Eap1p is involved in the decapping of Vts1p target transcripts we made use of an antibody that recognizes the mRNA 5′ cap structure. A transcriptional pulse-chase experiment was performed and RNA harvested 16-min post glucose addition was immunoprecipitated with the 5′ cap antibody. The levels of *GFP-SRE^+^* mRNA in the immunoprecipitates and in the flow through were assayed via Northern blot to provide a quantitative measure of the percentage of capped versus uncapped mRNA. The results from *eap1Δ* cells were compared to results obtained from cells depleted of Dcp2p, which is the catalytic subunit of the decapping complex, and *xrn1Δ* cells, which are defective for 5′-to-3′ exonucleolytic decay. Depletion of Dcp2p was achieved using a strain expressing *DCP2* under a tetracycline regulatable promoter (TetO_7_-*DCP2*), where inhibition of *DCP2* transcription results from addition of the tetracycline analog doxycycline [27].

**Figure 5:**
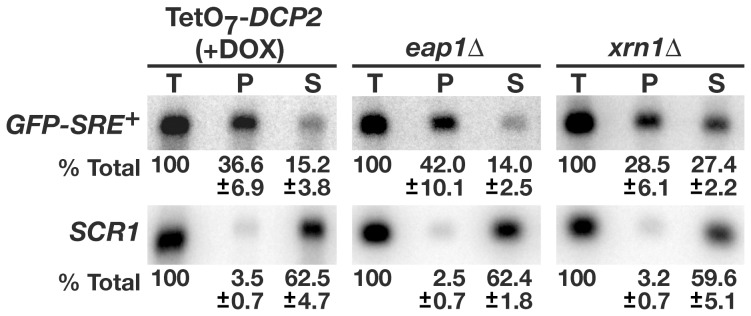
Eap1p stimulates transcript decapping. Total RNA was isolated from the indicated cells expressing the *GFP-SRE^+^* reporter during transcriptional pulse-chase experiments 16 min after transcriptional shut off with glucose. These samples were subjected to immunoprecipitation using an antibody that recognizes the 5′ cap and equivalent amounts of RNA from starting material (T), immunoprecipitated material (P) and the supernatant from the immunoprecipitates (S) were assayed for *GFP-SRE^+^* mRNA and *SCR1* RNA levels via Northern blots. The results of three independent experiments were quantitated and the average percentage of material in each sample along with the standard deviation are indicated. Note that *GFP-SRE^+^* is significantly enriched in immunoprecipitates from both TetO_7_-*DCP2* cells treated with doxycycline and *eap1Δ* cells as determined by a t test (P<0.01) while this is not the case in immunoprecipitates from *xrn1Δ* cells.

In these experiments we recovered on average ∼50% of the *GFP-SRE^+^* mRNA. When cells were depleted of Dcp2p and therefore defective for decapping, *GFP-SRE^+^* mRNA was enriched 2.4 fold in the immunoprecipitated material, with 36.6% of the total *GFP-SRE^+^* mRNA present in the immunoprecipitated fraction and 15.2% present in the supernatant (Figure 5). In contrast, *GFP-SRE^+^* mRNA was not enriched in the immunoprecipitated material from *xrn1Δ* cells, with similar levels of *GFP-SRE^+^* mRNA found in immunoprecipitated and supernatant fractions. Control experiments confirm the specificity of the antibody for the 5′ cap structure as ≤3.5% of the total *SCR1* RNA, which is an RNA polymerase III transcript that lacks a cap structure [28], was precipitated in any experiment. In *eap1Δ* cells, *GFP-SRE^+^* mRNA was enriched 3 fold in immunoprecipitated material, with 42% found in the immunoprecipitated fraction and 14% in the supernatant. The similar enrichment of *GFP-SRE^+^* mRNA in Dcp2p depleted cells and *eap1Δ* cells indicates that Eap1p is required for efficient decapping of *GFP-SRE^+^* mRNA.

### Eap1p Mediates an Interaction between Vts1p and eIF4E

**Figure 6:**
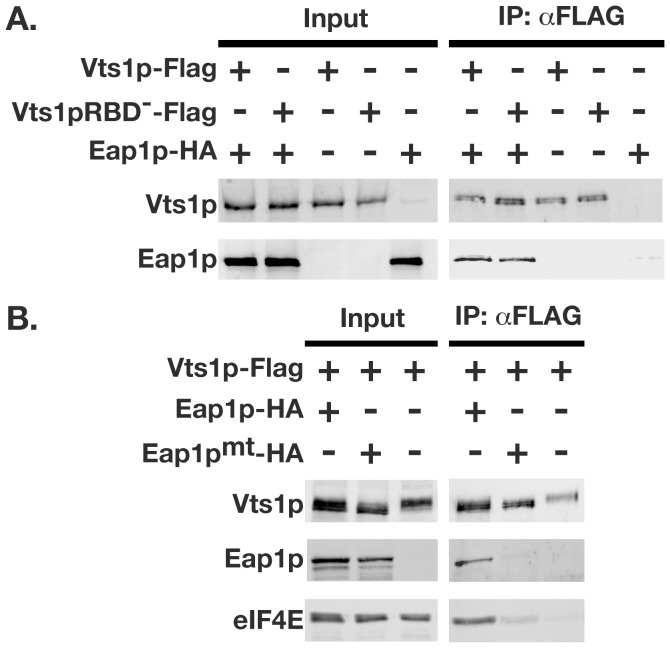
Eap1p mediates an indirect interaction between Vts1p and eIF4E. Vts1p-Flag and Eap1p-HA were expressed in yeast cells individually or in combination as indicated in either *vts1Δ* cells (**A**) or *eap1Δ* cells (**B**) and crude extracts were subjected to immunoprecipitation using an anti-Flag resin. Starting crude extracts (input) and the resulting immunoprecipitates (IP:αFLAG) were analyzed by Western blot. Input samples represent 5% for Vts1p blots, 2% for Eap1p blots and 1% for eIF4E of material used in the immunoprecipitations. The use of the Vts1p RNA binding mutant (Vts1pRBD^−^) and Eap1p mutant defective for eIF4E binding (Eap1p^mt^) are as indicated.

Given the requirement of Eap1p for Vts1p-mediated mRNA decay, we sought to determine whether Vts1p physically interacts with Eap1p in vivo. We performed co-immunoprecipitation experiments using whole-cell lysates from cells expressing Flag-tagged Vts1p and HA-tagged Eap1p. Vts1p-Flag was immunoprecipitated using anti-Flag resin, and the immunoprecipitates were analyzed by Western blot. Eap1p-HA was present in the anti-Flag immunoprecipitate when the Vts1p-Flag protein was present (Figure 6A). As a control, Eap1p-HA was not immunoprecipitated from an extract lacking Vts1p-Flag. The co-immunoprecipitation of Vts1p with Eap1p was RNA independent, as it was observed when Vts1p harbored an amino acid change (A498Q) that blocks its ability to bind RNA (Vts1pRBD^−^) [12].

The interaction of Vts1p with the eIF4E-binding protein Eap1p led us to hypothesize that Eap1p may mediate an interaction between Vts1p and eIF4E. To test whether Vts1p interacts with eIF4E via Eap1p we repeated our co-immunoprecipitation experiments using whole cell lysates derived from *eap1Δ* cells. Consistent with data presented above, Eap1p-HA was present in the anti-Flag immunoprecipitate when the Vts1p-Flag protein was present (Figure 6B). eIF4E was also detected in Vts1p-Flag immunoprecipitates only in the presence of Eap1p. eIF4E was not significantly enriched when Vts1p-Flag was immunoprecipitated from lysate expressing Eap1p that harbored amino acid changes (Y109A;L114A) in the eIF4E-binding motif that block its ability to bind eIF4E (Eap1p^mt^) [29]. In addition, we did not detect a significant interaction between Eap1p^mt^-HA and Vts1p-Flag, suggesting that binding of Eap1p to eIF4E may stabilize the Vts1p/Eap1p interaction. Taken together these data indicate that Eap1p mediates an interaction between Vts1p and eIF4E through its eIF4E-binding motif.

### Binding to eIF4E is Required for Eap1p to Stimulate Transcript Decay

**Figure 7:**
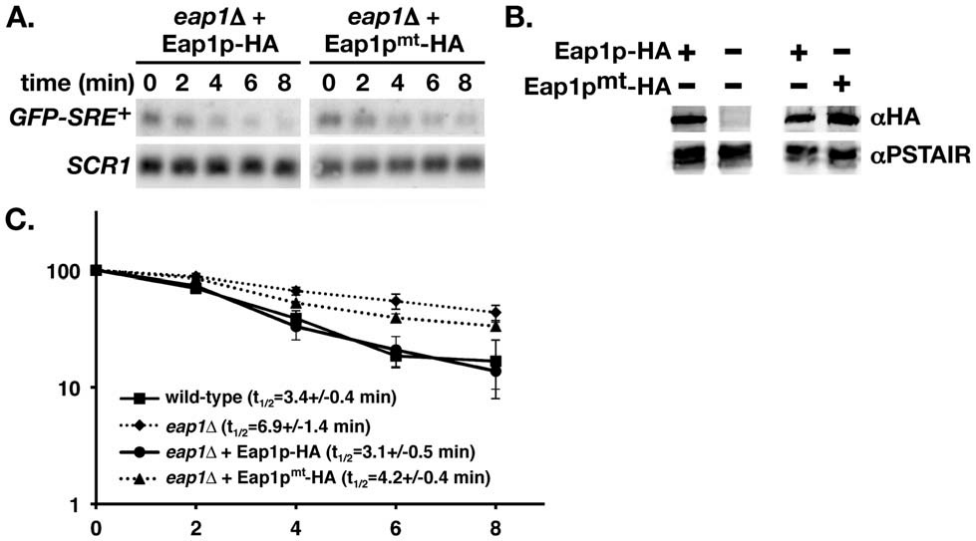
The Eap1p/eIF4E interaction is required for efficient decay of Vts1p target mRNAs. *eap1Δ* cells were rescued with either wild-type Eap1p-HA or a mutant version of Eap1p that is defective in its ability to interact with eIF4E (Eap1p^mt^-HA) and the stability of *GFP-SRE^+^* mRNA was assessed in these cells using a transcriptional pulse-chase approach and Northern blot (**A**) as described in Figure 1. Western blot (**B**) was used to compare the expression of wild-type Eap1p-HA to that of Eap1p^mt^-HA using the PSTAIR protein as a loading control. The results of at least 3 independent experiments were quantitated and normalized using the levels of *SCR1* RNA and graphed with error bars representing standard deviation (**C**). Data from wild-type and *eap1Δ* cells is from Figure 1A.

To explore the molecular mechanism that underlies the role of Eap1p in the decay of Vts1p target transcripts we asked whether the Eap1p/eIF4E interaction was required for mRNA degradation. This involved assaying the stability of *GFP-SRE^+^* mRNA by transcriptional pulse-chase in *eap1Δ* cells expressing either wild-type Eap1p (Eap1p-HA) or a version of Eap1p harboring the Y109A;L114A mutations that disrupt its eIF4E-binding ability (Eap1p^mt^-HA) [29]. When Eap1p-HA was expressed in the *eap1Δ* strain, *GFP-SRE^+^* mRNA decayed at a rate similar to wild-type (Figure 7A and C). However when Eap1p^mt^-HA was expressed in the *eap1Δ* strain, the decay rate of *GFP-SRE^+^* mRNA was not rescued to the wild-type rate. Western blot analysis confirmed that both Eap1p-HA and Eap1p^mt^-HA were expressed at similar levels and therefore differences in protein expression cannot account for the observed difference in mRNA decay kinetics (Figure 7B). These data demonstrate that a functional Eap1p eIF4E-binding motif is required for the rapid degradation of *GFP-SRE^+^* mRNA.

## Discussion

Here we demonstrate a role for Eap1p in Vts1p-mediated mRNA decay. The ability of Eap1p to interact with the cap binding protein eIF4E is required for this function and, consistent with its proximity to the 5′ cap, Eap1p stimulates mRNA decapping. While Eap1p is required for efficient decay of a reporter mRNA bearing wild-type SREs, it is not required for degradation of a reporter bearing mutated non-functional SREs. This suggests a specific role for Eap1p in the degradation of Vts1p target transcripts. In addition, we show that Vts1p interacts with Eap1p and that Eap1p is able to mediate an indirect interaction between Vts1p and eIF4E. Taken together these data suggest a model whereby recruitment of Eap1p to target transcripts, through its interaction with Vts1p, stimulates decapping via binding to eIF4E. Interestingly, another RNA binding protein, Puf5p, also functions with Eap1p to enchance the decapping of its target mRNAs (Aaron Goldstrohm, University of Michigan, personal communication).

While Eap1p has a role in the rapid decay of Vts1p target transcripts it does not appear to play a role in regulating their translation. For example, we have been unable to document a role for Vts1p in regulating translation that is independent of transcript decay. For example, we have shown that GFP protein levels from the *GFP-SRE^+^* mRNA are repressed approximately four fold compared to *GFP-SRE^-^* mRNA and that this repression is eliminated in a *vts1Δ* strain ( [12], and data not shown). However, when GFP protein levels were corrected using the levels of GFP mRNA we did not see a statistically significant difference in the ratio of GFP protein levels from the GFP-SRE*^+^* and *GFP-SRE^-^* mRNAs in wild-type, *vts1Δ* and *eap1Δ* cells (data not shown). These results suggest that repression of *GFP-SRE^+^* reporter expression is mediated largely through degradation of target mRNAs, however we cannot rule out some role for translational repression mediated by Vts1p and/or Eap1p that our assays are not sensitive enough to detect. Alternatively, since the poly(A) tail is known to stimulate translation, Vts1p-mediated deadenylation of target mRNAs could simultaneously repress translation and induce transcript decay; if so, the two processes would not be separable.

A lack of a role for Eap1p in translational repression of Vts1p target mRNAs is consistent with the fact that a genome-wide survey of mRNAs that are translationally regulated by Eap1p show no statistically significant overlap with mRNAs that are bound by Vts1p [30]. Thus the pool of Eap1p target mRNAs that are translationally regulated are distinct from the pool of Vts1p target mRNAs that are degraded. Since a poly(A) tail is required for mRNA translation, perhaps Vts1p-mediated deadenylation represses mRNA translation to the point where Eap1p can no longer augment this effect. In contrast, Eap1p is able to enhance decapping mediated by Vts1p-mediated deadenylation.

### The Role of Eap1p in mRNA Decapping

What is the molecular mechanism that underlies the role of Eap1p in decapping? The mRNA cap structure stimulates translation through the ability of eIF4E to recruit the translation machinery to an mRNA through the eIF4E/eIF4G interaction. A critical step in mRNA decapping is thought to involve removal of the translation initiation complex from the mRNA. This model is based on results arguing that the decapping and translation machineries compete with one another for access to mRNAs [31], [32]. For example, disruption of eIF4G function in yeast results in increased rates of decapping [31]. We find that the eIF4E-binding motif in Eap1p is required for its role in Vts1p-mediated degradation. Thus the eIF4E/Eap1p interaction may enhance decapping by destabilizing the translation initiation complex through displacement of eIF4G from the mRNA.

Eap1p may also enhance decapping by facilitating the recruitment of decapping factors to the 5′ end of the mRNA. This is supported by systematic efforts that identified protein complexes in *S. cerevisiae*, including an interaction between Eap1p and the decapping activator Dhh1p [33]. In this model the Eap1p/eIF4E interaction would serve to recruit Dhh1p to the vicinity of the cap thereby facilitating Dhh1p-mediated decapping. However this model may not be applicable to Vts1p target mRNAs as Vts1p-mediated decay of the *GFP-SRE^+^* mRNA does not involve Dhh1p [18].

### The Function of Eap1p in Yeast

Eap1p functions in a variety of biological processes including pseudohyphal growth [29], the response to oxidative stress induced by exposure to diamide and cadmium [34], the response to vesicular transport defects [35] and the regulation of rapamycin-induced inactivation of TOR-signaling [25], [36]. In addition, polysome analysis has shown that Eap1p regulates the translation of specific target transcripts [29]. Taken together with our data, these results suggest that Eap1p regulates the translation and/or stability of specific mRNAs under a variety of physiological conditions and that its interaction with RNA-binding proteins such as Vts1p (this study), Puf1 and Puf2 [30] as well as Puf5 (Aaron Goldstrohm, University of Michigan, personal communication), at least in part, controls which transcripts Eap1p regulates.

### The Role of eIF4E-binding Proteins in mRNA Decay

Another eIF4E-binding protein, known as 4E-transporter (4E-T), has been shown to play a role in transcript decay in mammalian cells [37]. Originally identified as being responsible for transporting eIF4E into the nucleus [38], subsequent experiments showed that depletion of 4E-T from HeLa cells resulted in significant stabilization of A/U rich element (ARE)-containing mRNAs [37]. It is thought that 4E-T may regulate the transition from translational repression to mRNA degradation through a mechanism involving the localization of mRNAs to P-bodies, which are sites of mRNA storage and decay [37], [39]. While the role of the 4E-T/eIF4E interaction in ARE-mediated decay has not been assessed, indirect evidence suggests that 4E-T may also stimulate decapping. Though both are eIF4E-binding proteins, Eap1p and 4E-T are not homologs. Thus, these data suggest that other eIF4E-binding proteins might play a role in stimulating transcript decapping through their interaction with eIF4E.

## Materials and Methods

### Yeast Strains and Media

Yeast strains used in this study were derivatives of the wild-type BY4741 [40]. All deletion strains are as described by Winzeler et al., (1999), with the exception of the *eap1Δ* strain which was generated for this study by directed PCR-mediated gene deletion (*MAT* **a** *eap1Δ::KANMX6 leu2Δ0 his3Δ1 ura3Δ0 met15Δ0*). The TetO_7_-*DCP2* strain is described in Mnaimneh et al., (2004). The strains were transformed by standard techniques, and plasmids were maintained by growth in selective media.

### mRNA Analysis

Transcriptional pulse-chase experiments employed *GFP-SRE^+^*, GFP-SRE*^-^*, and GFP-YIR016W reporters described in Rendl et al., [18]. For these experiments cells were grown in selective medium containing 2% raffinose at 30°C to mid-log phase and then cooled to 20°C. Due to the short half-life of the *GFP-SRE^+^* reporter in wild-type cells, an accurate measure of reporter mRNA stability at 30°C was not possible. Cooling cells to 20°C slowed the decay of *GFP-SRE^+^* mRNA to allow for measurement of reporter mRNA half-lives. GFP reporter transcription was initiated by the addition of galactose to a final concentration of 2% for 16 min and then repressed by the addition of glucose to a final concentration of 4%. Total RNA was isolated by glass bead lysis in LET buffer (100 mM LiCl, 20 mM EDTA, 25 mM Tris-HCl at pH 8.0) and LET-equilibrated phenol at the indicated time points and analyzed by Northern blot. *SCR1* or *ACT1* RNA was used for normalization of transcript levels as indicated.

RNase H cleavage assays were performed as described by Decker and Parker (1993), using an oligonucleotide that hybridized ∼330 nt upstream of the GFP reporter poly(A) site. To control for differences in RNA concentration a portion of each RNA sample was analyzed via a standard Northern blot and probed for *SCR1* RNA. Northern blots were exposed to PhosphorImager screens and analyzed with ImageQuant Software.

For RNA immunoprecipitations total RNA was isolated from cells expressing the *GFP-SRE^+^* reporter during transcriptional pulse-chase experiments 16 min after transcriptional shut-off with glucose. Immunoprecipitations were performed as described in Muhlrad et al. (1994) using monoclonal anti-m_3_G-/m^7^G-cap (Synaptic Systems) with the following modifications. Anti-m_3_G-/m^7^G-cap antibodies were prebound at 4°C to protein G-Agarose (Roche) in IPPH (500 mM NaCl, 10 mM Tris-Cl at pH 7.5, 0.1% NP-40) for 2 h. The beads were extensively washed in IPPL (150 mM NaCl, 10 mM Tris-Cl at pH 7.5, 1 mM EDTA, 0.05% NP-40) and then incubated at 4°C with 5 µg total RNA in IPPL supplemented with 1 mM DTT and 0.1 U/µL RNasin (Promega) for 2 h. The supernatant was recovered and incubated for 2 h at 4°C a second time with antibody prebound to beads. The pellets were washed twice with IPPL and combined. Bound and unbound mRNA was then isolated from the pellet and supernatant fractions and analyzed by Northern blot.

For Figure 7 transcriptional pulse-chase experiments were performed to analyze reporter mRNA stability as described above. For analysis of protein expression a sample of cells at time 0 were denatured by trichloroacetic acid (TCA) precipitation, lysed with glass beads and the resulting extracts fractionated on SDS-PAGE and analyzed by Western blot using HA antibodies (Santa Cruz) and PSTAIR antibodies (Sigma) as indicated.

### Immunoprecipitations

Immunoprecipitation was performed essentially as described previously in Rendl et al. (2008). The plasmid expressing Vts1p-Flag was generated in pRS315 with a C-terminal Flag tag and 574 bases of genomic sequence upstream and 396 bases downstream of the *VTS1* ORF. The plasmid expressing Vts1pRBD^–^Flag was generated in pRS315 and is identical in sequence to Vts1p-Flag with the exception of the A498Q amino acid change. All Vts1p constructs also harbor VSV epitopes that were used for protein detection by Western blot. The Eap1p-HA plasmid was created in pRS316 with a C-terminal 3X HA tag and 490 bases of genomic sequence upstream and 492 bases downstream of the *EAP1* ORF. The Eap1p^mt^-HA construct was generated in pRS316 and is identical in sequence to Eap1p-HA with the exception of the Y109A;L114A amino acid changes. Preparation of protein extracts by glass bead lysis and subsequent immunoprecipitation experiments were preformed as previously described in Rendl et al. (2008). Briefly anti-Flag M2 affinity gel (Sigma) was used to immunoprecipitate Flag-tagged proteins for 3 h at 4°C. After extensive washing Vts1p-Flag and associated proteins were eluted with Flag-peptide (Sigma). VSV antibodies (Bethyl Laboratories), HA antibodies (Santa Cruz) and eIF4E antibodies (a generous gift from N. Sonenberg [25]) were used to detect eluted proteins by Western Blot.

## Supporting Information

Figure S1 ***GFP-SRE*** *^-^* **mRNA is degraded through a Ccr4p/Xrn1p-dependent pathway.** *GFP-SRE^-^* gene transcription was induced in wild-type, *ccr4Δ* and *xrn1Δ* cells with galactose and then shut off with glucose and reporter mRNA levels were assayed at the times indicated after transcriptional shutoff by Northern blot. The results of at least two independent experiments were quantitated and normalized using the levels of *SCR1* RNA and graphed with error bars representing standard deviation. Note that there is signficantly less *GFP-SRE* ^-^ mRNA in wild-type cells compared to either *ccr4Δ* or *xrn1Δ* cells at the 30 minute time point (P<0.03).
(TIF)

Figure S2 ***GFP-SRE*** *^+^* **and** ***GFP-SRE*** *^-^* **mRNAs have the same stability in** ***vts1*** *Δ* **cells.** *GFP-SRE^+^* and *GFP-SRE^-^* gene transcription was induced in *vts1Δ* cells with galactose and then shut off with glucose and reporter mRNA levels were assayed at the times indicated after transcriptional shutoff by Northern blot. The results of at least two independent experiments were quantitated and normalized using the levels of *SCR1* RNA and graphed with error bars representing standard deviation. Data for *GFP-SRE^-^* is from Figure 3.
(TIF)
