# Supplementary figures and images for: The eIF4E-Binding Protein Eap1p Functions in Vts1p-Mediated Transcript Decay
Source: PLoS One. 2012 Oct 10;7(10):e47121. doi: 10.1371/journal.pone.0047121 (PMC3468468; doi:10.1371/journal.pone.0047121)

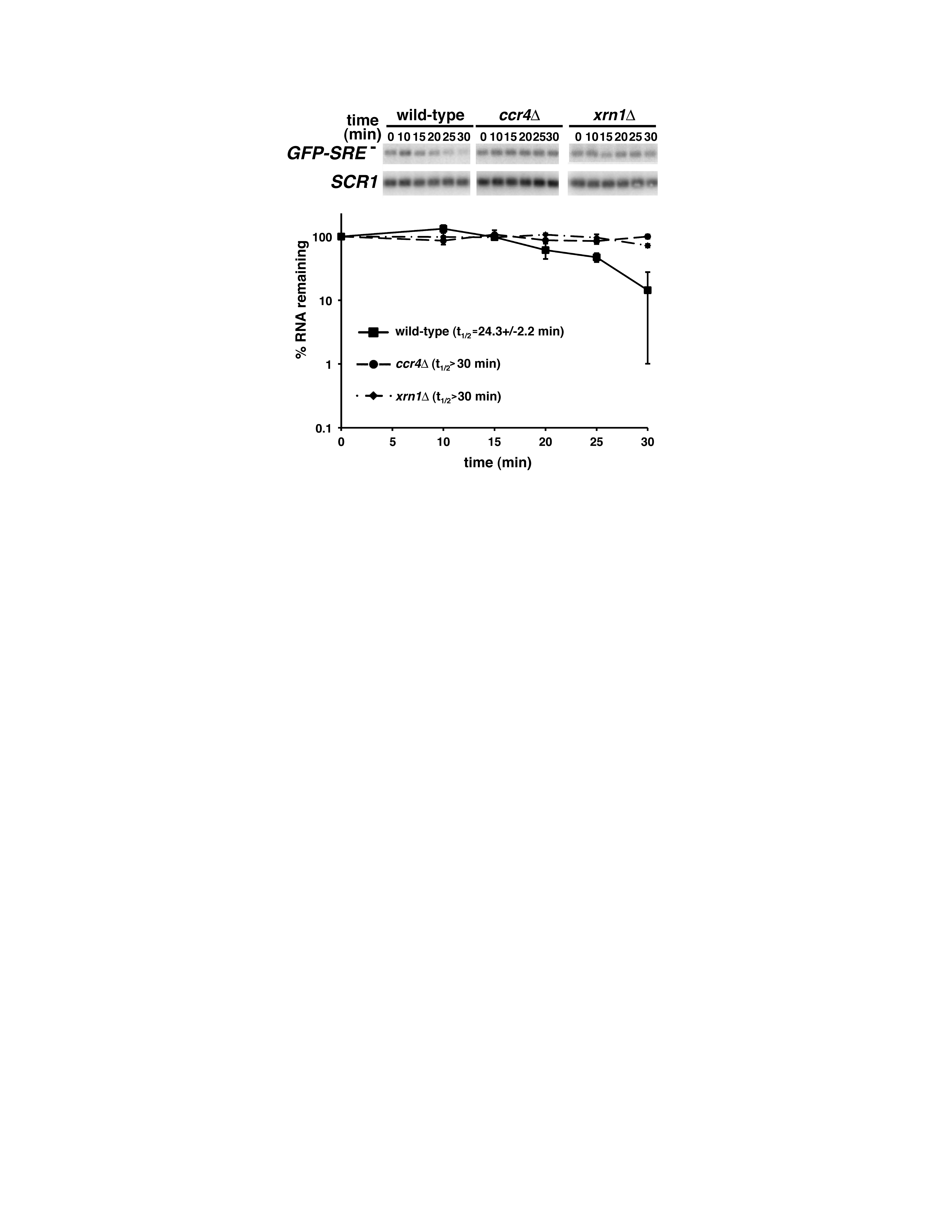

Supplement: Figure S1 — GFP-SRE - mRNA is degraded through a Ccr4p/Xrn1p-dependent pathway. GFP-SRE- gene transcription was induced in wild-type, ccr4Δ and xrn1Δ cells with galactose and then shut off with glucose and reporter mRNA levels were assayed at the times indicated after transcriptional shutoff by Northern blot. The results of at least two independent experiments were quantitated and normalized using the levels of SCR1 RNA and graphed with error bars representing standard deviation. Note that there is signficantly less GFP-SRE - mRNA in wild-type cells compared to either ccr4Δ or xrn1Δ cells at the 30 minute time point (P<0.03). (TIF) [file pone.0047121.s001.tif]

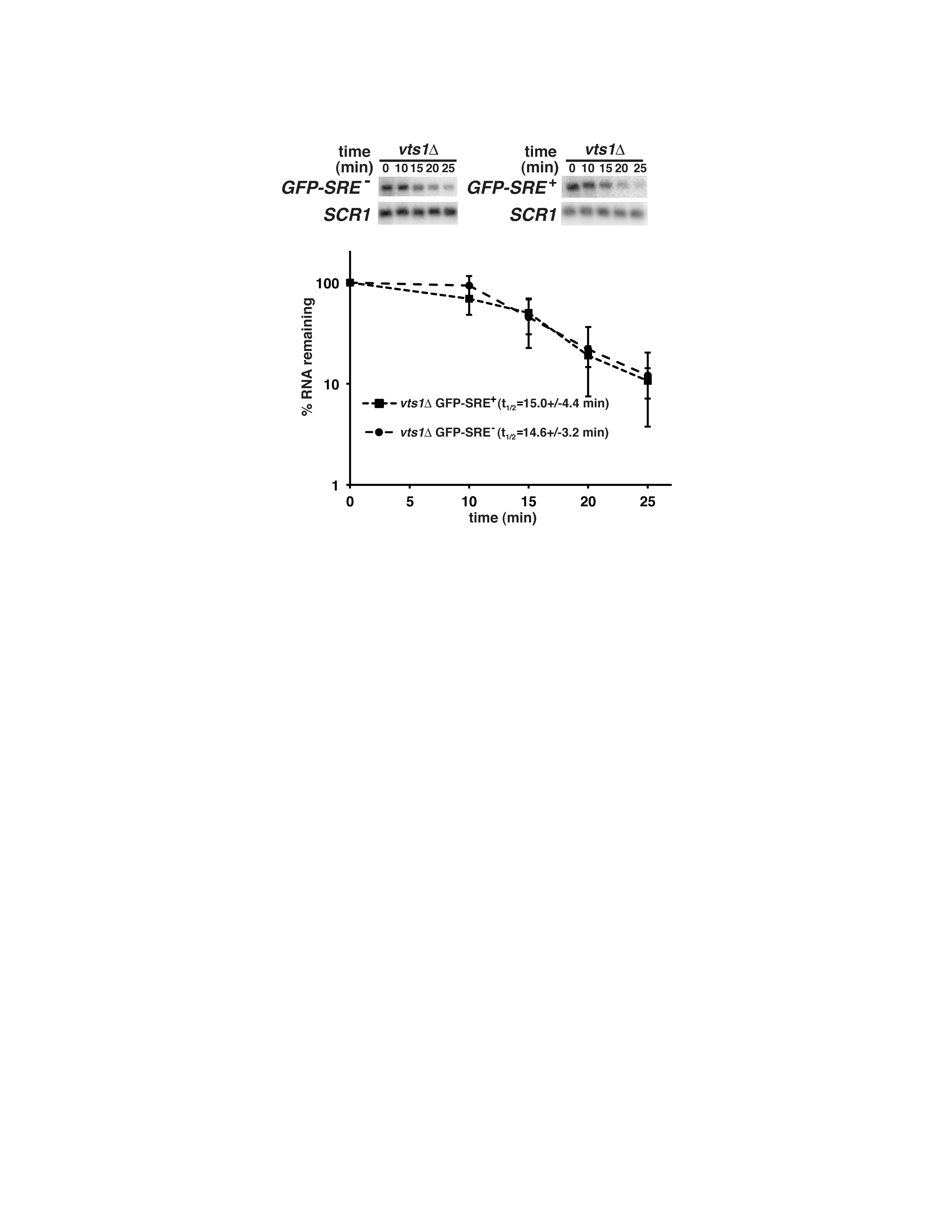

Supplement: Figure S2 — GFP-SRE + and GFP-SRE - mRNAs have the same stability in vts1 Δ cells. GFP-SRE+ and GFP-SRE- gene transcription was induced in vts1Δ cells with galactose and then shut off with glucose and reporter mRNA levels were assayed at the times indicated after transcriptional shutoff by Northern blot. The results of at least two independent experiments were quantitated and normalized using the levels of SCR1 RNA and graphed with error bars representing standard deviation. Data for GFP-SRE- is from Figure 3. (TIF) [file pone.0047121.s002.tif]
